# Equity, diversity, and inclusion in oncology pharmacy practice: Everyone's business

**DOI:** 10.1177/10781552241264717

**Published:** 2024-07-23

**Authors:** Pascale Dettwiller, Suhani Ghiya, Jurga McLean, Stewart O’Callaghan, AdeDolapo Sanni, Nisha Shaunak, Lilia So, Steve Williamson, Shereen Nabhani-Gebara

**Affiliations:** 1 Pharmacy Department, Department of Rural Health, 1067 The University of South Australia, Port Lincoln, South Australia, Australia; 2 6979 Pharmacy Department, The Rotherham NHS Foundation Trust, Rotherham, South Yorkshire, UK; 3 4970 Pharmacy Department, The Royal Marsden NHS Foundation Trust, London, UK; 4 Public Private Partnership, OUTpatients, 4468 University of Leeds, London, UK; 5 Pharmacy Department, 15, Cornfield close, Sittingbourne, Kent, UK; 6 Pharmacy Department, Guy's and St Thomas’ NHS Foundation Trust, London, UK; 7 13985 National Institute for Health and Care Excellence (NICE), London, UK; 8 Pharmacy Department, Faculty of Health, Science, Social Care & Education, Kingston University, London, UK

**Keywords:** equity, diversity, inclusion, pharmacy practice, oncology, policy change

## Abstract

**Introduction:**

Equity, Diversity, and Inclusion (EDI) is gaining increased attention within all industries healthcare being no exception. The terminology Equity, Diversity, and Inclusion and its abbreviation EDI gained popularity in the early 2000's when varied socio-political factors prompted many organisations to examine EDI concepts and how to operationalise them. The growing diversity of our society requires cross-cultural inclusive approaches to increase equity and access to services.

**Method:**

This unique research is community-led research supported by the British Oncology Pharmacy Association, in which the members of the BOPA community are equal partners to inform action on policies that address EDI. This research was a cross-sectional study involving an online survey of financial BOPA members.

**Results:**

Demographic data was extracted, and the quotes were analysed for common themes. The majority of respondents were women, and the largest age group was between 34 and 44. The first cause of microaggressions identified by the respondents was of racial and ethnic origin, followed by marital status and religious nature. Participants described the lack of diversity in senior positions and the microaggressions experienced by those who hold leadership positions. Some participants described how some situations at work made them feel excluded or alienated. The impact of discrimination and bullying/microaggressions extended to patients was also reported.

**Conclusion:**

Despite strategic directions encompassing this aspect, this research underscores the pressing need for more evidence on the lack of EDI in healthcare institutions. Our findings, located in the pharmacy oncology specialty, have identified the problem and highlighted the potential benefits of addressing it. More needs to be done in training and professional development to address unconscious bias and change behaviours.

## Introduction

Very little evidence exists about healthcare professional's experiences and understanding of equity, diversity, and inclusion (EDI) principles in the workplace. The pharmacy profession has been quite discreet in acknowledging and responding to challenging behaviours related to the breach of EDI principles in the work environment. Crises such as the COVID-19 pandemic have brought to national attention the dire consequences of failing to provide accessible, equitable care for all individuals in our society.^1^ Diversity is about similarities and differences among individuals that comprise personality and identity; equity is fair practices in access, opportunities, and advancement, and inclusion fosters a sense of value, respect, and empowerment in employees.^2^

EDI is gaining increased attention within all industries healthcare being no exception. Universities seem to have led awareness and actions in the late 1960s when traditional social relations and educational practices were being challenged the world over; Carnegie Mellon University (in Pittsburgh, Pennsylvania, USA) started making significant changes in its organisation.^2^ In the intervening years, the institution launched several programs with diversity as its goal. The terminology Equity, Diversity, and Inclusion and its abbreviation EDI gained popularity in the early 2000's when varied socio-political factors prompted many organisations to examine EDI concepts and how to operationalise them. The growing diversity of our society requires cross-cultural inclusive approaches to increase equity and access to services. The actual value of diversity has been increasingly understood and appreciated via data that matured in the 1990s and 2000s, mainly from the corporate world.^3^ Workplace diversity training emerged in the mid-1960s following affirmative action and equal employment laws put in place around the globe. The principle of EDI is addressing inequities beyond racial and gender discrimination.

Many job seekers significantly consider a company's diversity when applying and this extends to various sectors: academia, pharmaceutical industry, and clinical workforce.^4–6^

There is a lack of research to enhance our understanding of the current climate specifically in pharmacy practice. In 2020, Patel et al.^1^ were one of the pioneers that raised workforce issues in the context of EDI in a policy statement from the American Society of Clinical Oncology. Patel et al. recognised that achieving health equity among healthcare professionals requires efforts from all individuals, general public, policymakers, health systems, and other stakeholders. One of the recommendations to promote health equity describes the importance of promoting and improving cancer workforce diversity. Therefore, it is important to consider the experience and wellbeing of healthcare professionals within diverse environments in an effort to support sustainability and a healthy working environment for all. Patel et al. also highlighted the need to address institutional discrimination by facilitating open forums at meetings and webinars, and hence raising awareness and action that address health inequities.

This exploratory research was supported by the British Oncology Pharmacy Association (BOPA) executive committee, which established an EDI subcommittee in 2022 to focus on EDI as principles guiding the organisation's strategies and supporting its membership in an area of increasing significance for the healthcare industry. This work is community-led research in which the members of the BOPA community are equal partners to inform action on policies that address EDI. It focuses on understanding the membership awareness of EDI principles in their workplaces and gathering members’ stories to evidence inequalities and enable the committee to identify areas for training.

The survey results will be used to help healthcare institutions develop robust policies, not just training to address implicit bias. The results also assist collaboration with other organisations working in the same space.

## Method

The Checklist for Reporting Results of Internet E- Surveys (CHERRIES checklist) was used to describe the adopted methodology.^7^

This was a cross-sectional study involving an online survey of BOPA members.

Due to the dearth of literature exploring EDI in the pharmacy profession, a small working party, including a patient representative, designed a new instrument using human resources exemplars from business organisations. The tool is structured into four parts (40 questions): one exploring members’ EDI experiences at their current employing organisation using a 5-point Likert scale rating for the eleven statements; part two is related to BOPA as an association wanting to implement EDI, asking as well about topics for training and medium for the delivery (and will not be reported in this article), part three contains dimensions of diversity relevant to the study primarily age, gender identity, ethnicity, and secondary disability and region; and at the end part four is made up of two open-ended questions exploring EDI experiences as a witness and as personal lived experience. An option ‘*prefer not to answer’* was used to avoid individuals skipping the question. The instrument provided quantitative and qualitative data aligned with the choice of a mixed-method design for enriched data collection and to provide context to the qualitative part.

Face and content validation were completed with an independent group from the EDI subcommittee and the BOPA executive committee, leading to four iterations. Face validation assessed whether the questions were valid to the participants. Content validation was performed by experts and assessed whether the survey contains questions which cover all aspects of the construct being measured.

A SurveyMonkey SM® survey was created and administered®.^8^

The participants’ pool represented the financial members pharmacists and technicians working in community, academia, industry, and hospital settings. As this project was planned to be a descriptive study and not a representative one, the survey remained open for 4 months with five reminders sent to the membership by email and social media.

The survey was sent to the membership that was blinded by the EDI research working party. The online survey was disseminated before and after the 25^th^ annual conference in Liverpool. The survey was closed in mid-December 2022. By answering the survey, the respondents provided consent.

The data was exported from SM® into Microsoft® Excel spreadsheets for analysis, after which the responses were deleted from SM® platform. The open comments were screened and analysed for common themes by two independent researchers. Descriptive statistics were used to analyse the responses to the close-ended questions.

Even though the whole EDI committee contributed to the design of the survey, a small BOPA task and finish working group was created to analyse the data and finalise the survey results. The group was formed of five members; they have reviewed and evaluated the data.

Ethics approval process following university governance and approval by the executive committee were sought and granted (number: H-2023-019).

## Results

Ninety-one BOPA members participated in the study, representing 13.4% of the estimated current membership at the time. The response rate to all the questions is 94% across the questionnaire.

**Figure 1.**
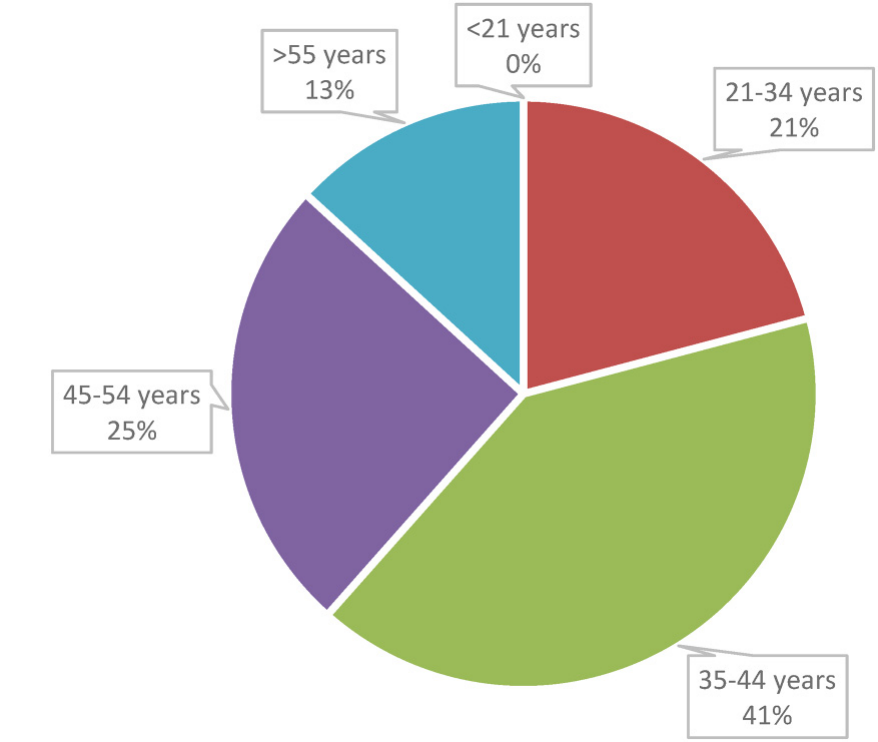
Distribution of respondents’ age.

Table 1 presents the primary and secondary demographics of the sample and Figure 1 shows the age distribution of the respondents. Most respondents were women, and the largest age group was between 34 and 44.

**Table 1.** Primary and secondary demographic and professional characteristics of surveyed BOPA members (when numbers do not add up to 91 – the difference represents ‘prefer not to say’ group).

Primary	Number (%) N = 91
Gender	
Female	64 (70)
Male	27 (30)
Ethnicity	
White	46 (50)
Asian	22 (24)
Black/African/Caribbean	5 (5)
Sexual orientation	
Heterosexual/straight	83 (92)
Gay/lesbian	1 (1.1)
Secondary	Number (%) N=91
Religion	
Christian	30 (33)
No religion	17 (19)
Muslim	13 (14)
Atheist	8 (9)
Hindu	7 (8)
Jewish	2 (2.2)
Buddhist	2 (2.2)
Sikh	1 (1.1)
Prefer not to say	7 (8)
Different needs	
Yes	7 (8)
**Length of employment with current employer**	
Ten years and more	29 (32)
Two years to less than 5 years	20 (22)
Five years to less than 10 years	18 (20)
Less than 1 year	18 (20)
One year to less than 2 years	6 (7)

Participants of the survey were provided with male and female gender choices as well as male trans and female trans; thus there was an opportunity to self-identify as a different gender. There was also an option ‘prefer not to say’ when choosing gender rather than ‘unsure’, which has no statistical use. When choosing ‘prefer not to say’ it implies that the respondent has actually made a choice and a decision aligned with their values. Choices for race and sexual orientation were preset in the survey; however, respondents were allowed to say ‘other’ and asked to specify if not listed as well as given an option to choose ‘prefer not to say’. As part of the consultation undertaken for the survey design, the research team decided to drift from the legal definition of special needs or disability by using the terminology of different needs to allow respondents to identify if those differences were addressed in the context of the EDI principles.

### Experiences with current employer

All respondents answered the questions relating to EDI experiences with the current employer with the highest agreement score pertaining to the statement ‘*I believe the organisation will take appropriate action in response to incidents of discrimination and/or bias incidents*’ and the highest disagreement related to the statement ‘*This organisation has done an excellent job providing educational programs that promote diversity, equity and inclusion in the workplace*’.

There was consistency in the answers with a low rate of disagreement (<9%).

Table 2 presents the participants’ rating on a Likert scale of 5 of statements exploring their experience of EDI as an employee.

**Table 2.** Respondents rating on a Likert scale of 5 of statement regarding employment and EDI.

	Strongly disagree	Disagree	Undecided	Agree	Strongly agree
I believe the organisation will take appropriate action in response to incidents of discrimination and/or bias incidents	6%	11%	17%	54%	12%
Employees appreciate others whose backgrounds, beliefs, and experiences are different from their own	2%	9%	18%	53%	18%
This organisation fosters a workplace that allows employees to be themselves at work without fear. e.g. commitment to meeting the needs of employees with disabilities	7%	15%	11%	52%	15%
The leadership at this organisation treats all employees fairly and provides an environment for the free and open expression of ideas, opinions, and beliefs.	7%	14%	16%	47%	15%
Employees of all background are treated fairly in the internal promotion process	8%	14%	22%	46%	10%
The leadership at my organisation encourages equity, diversity and inclusion through their processes and procedures e.g. awareness of the organisation's EDI policy and where this can be found.	3%	13%	23%	45%	13%
I feel confident challenging inappropriate behaviour not in line with EDI principles in my organisation	5%	19%	19%	43%	14%
Employees of different background are encouraged to apply for higher position	9%	10%	27%	41%	13%
The organisation is committed to improving the diversity of its employees through its recruitment process	4%	11%	34%	41%	10%
Management shows that diversity is important through its actions by discouraging biases and abuses	4%	16%	30%	36%	12%
This organisation has done an excellent job providing educational programs that promote diversity, equity, and inclusion in the workplace	5%	30%	24%	33%	8%

EDI: equity, diversity, and inclusion.

Of the eleven questions, nine (81%) rated between 50% and 70% for aggregated data ‘*agree*’ and ‘*strongly agree*’. In spite of that, 30–40% of all respondents did not agree (strongly agree and disagree) with these statements.

The ‘*neither agree nor disagree*’ responses vary between 11% and 34%, reflecting that some individuals might have heard of organisational processes but are unsure how EDI policies are implemented and how recruitment processes are fair and transparent. These comments will be evidenced in the analysis of the lived experiences comments.

Two questions of interest have a less-than-average agreement rating;
‘*This organisation has done an excellent job providing educational programs that promote diversity, equity and inclusion in the workplace’.* with only **33%** of respondents agreeing with the statement;

And,

‘*Management shows that diversity is important through its actions by discouraging biases and abuses’*. **36%** of respondents agreed with the statement.

Over half of the respondents (56%) consider recruitment processes as ‘fair’ and that promotion on merit is maintained in the organisations; ‘*employees of different backgrounds are treated fairly in the internal promotion process’.*

Sixty-seven per cent of the respondents indicated that ‘*their organisation will take appropriate action in response to discrimination and bias incidents’*.

**Figure 2.**
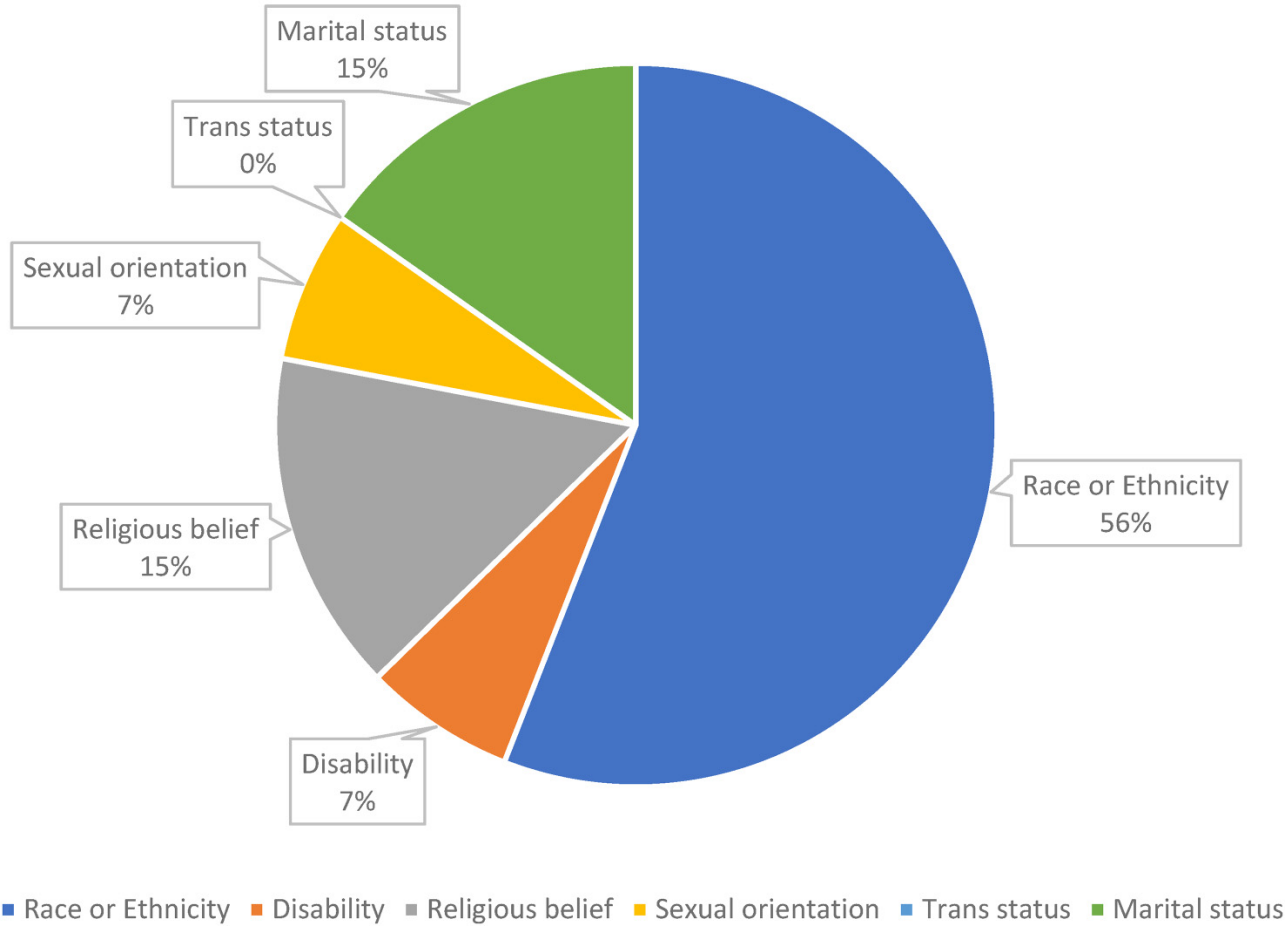
Responses of possible microaggressions reasons.

When asked about negative experiences relating to ‘discrimination’, the answers were balanced, with 45% (n = 41) responding YES and 49% (n = 45) NO. Five members (5%) preferred not to answer the question. In the following question, microaggressions in the workplace were explored, and 41% (n = 37) responded YES and 55% (n = 50) NO. Four people (4%) preferred not to answer the question. When asked about the perceived reasons for the microaggression, 56% of the respondents (n = 33/59) believe that the origin of the microaggression(s) is racial/ethnic. At the same time, 15% described the origins as due to marital status and religious beliefs. The other causes were sexual orientation (7%) and disability (7%), (Figure 2).

Data from the open responses enriched the quantitative findings and provided context. Of the participants who added comments, most reported a negative work experience. Table 2 provides a selection of quotes that further represent the identified themes.

### Discrimination at work

Participants experienced discrimination at work ranging in intensity, frequency, and impact. Some of the discrimination related to career development opportunities and recruitment while others impacted the feeling of belonging to the team.

### Bullying and microaggressions at work

Participants openly shared their experience of bullying and microaggressions relating to gender, ethnicity, religion, region, and marital status and whether they have a young family.

They also expressed their frustration with the lack of support and the dismissive culture and not acting upon complaints.

### Lack of equity and diversity

Participants described the lack of diversity in senior positions and the microaggressions experienced by those who hold leadership positions.

### Feeling of othering

Some participants described how some situations at work made them feel excluded or alienated.

### Patients

Even though this study aimed to explore staff experiences, a theme emerged among several responses, explaining that patients also have experienced microaggressions and were treated differently because of their skin colour or foreign accent.

### Parents

Participants who were parents described the difficulties and discrimination encountered when trying to juggle the responsibilities of a young family and their professional aspirations.

### Disability

Participants with additional needs described the inequity that results from lack of adjustments at work (Table 3).

**Table 3.** Themes and quotes.

Theme	Quotes
**Discrimination at work**	‘*Segregation from particular cohort due to my ethnicity in a subtle way and by exclusion from social events’*. (#61)
	‘*favouritism based on colour and ethnicity within the workplace. not being involved in development opportunities and chances for promotion’*. (#65)
	‘*As a British Asian I have to work harder to be valued for my knowledge and expertise especially when dealing with white MDT members’*. (#39)
	‘*I was told in confidence by someone on the hiring committee of a job I did not get that me self and a male candidate were both more than capable to do the job but the person with the final say wanted a man in the role as he was sick of working with women’*. (#27)
	*‘Unequal opportunities for career progression. Bullying, intimidation and harassment in the workplace. Inappropriate comments about religious belief. Abuse of power and authority’.* (#87)
**Bullying and microaggressions at work**	‘*Was very difficult made me poorly, nobody helped me to tackle the bullying, tried to fight it on my own’*. (#31)
	‘*Concerns were raised with senior pharmacy leadership regarding behaviour of a member of staff who made remarks such as “shipping you off” in reference to three members of staff from ethnic minority backgrounds being transferred to another site. This has racial connotations to slavery. Sadly, management continued defended her and in fact rebuked a colleague who raised concerns’.* (#59)
	‘*Not being made to feel welcomed, dismissal of ideas, feeling belittled in front of senior colleagues’.* (#52)
	‘*Manager has mocked my religions practices as I’m not a devout follower. Annual leave considerations especially during school holidays and Christmas favour those with children’*. (#39)
	‘*Colleagues openly making derogatory comments such as “people from your country don’t tend to succeed”. Managers making remarks during the holy month of Ramadan and observing fasting that they feel it's unsafe and disagree with not being to drink water etc’. (#42)*
**Lack of equity and diversity**	‘*No people of colour in management positions in this organisation’* (#7)
	‘*One consultant complaining of the smell of another consultant who was African’.* (#29)
**Feeling of ‘Othering’**	‘*The oncall rota needed a volunteer due to sickness. Most of the band 6 pharmacists were planning to go to a wine tasting event on the day of the gap. Two pharmacists of a religious belief/ethnicity that do not drink alcohol were approached to do the on call rather than the whole group’. (#48)*
**Patients**	‘*Patients with tanned skin are treated differently at my regional hospital - lack of awareness of diversity and service available to assist those groups; not using health literacy language that the patient can understand and assume that nodding the head is a sign of acquiescence, making inappropriate comments at the nursing station on relation to body odour, cleanliness of clothing, and smoking habits. it is difficult to call it out without witnessing the whole interaction. I tried to talk to some health care professional about his inappropriate behaviours but I feel it has fallen in a deaf ear. Management reassured me that more education will be provided in the future; those patients abscond the acute services and represent with more severe symptoms’.* (#77)
	‘*Was told by a patient that they don't want to be treated by foreign scum’. (#82)*
**Parents**	‘*I was asked about plans for more babies by my manager after returning from maternity leave’. (#12)*
	*‘Discrimination in career progression due to flexibility required as having young family’.* (#50)
	‘*I have to really fight to get reducing hours, after maternity’*. (#63)
**Disability**	*‘Materials, including training materials, that were not accessible due to disability’*. (#66)
	‘*Questions raised about my competence and knowledge not linked to my actual competence and knowledge but linked to my disabilities and health conditions’. (#74)*
	‘*My disability has never been accommodated by organisations I have worked for. Despite suggesting standards for accessibility being provided to those preparing or presenting material, these have never been enforced. As such I continue to have a poorer experience, be disadvantaged by this lack of accommodation/ consideration. I always feel my disability is not considered as being “real” or that I have to find my own solutions. This means I feel excluded or prevented from having an equal opportunity to contribute to discussions of the content’*. (#66)

## Conclusions

This is the first study to explore the experience of oncology pharmacy professionals with EDI. The survey responses provided a detailed insight into the reality of EDI for oncology health pharmacy professionals.

### Microaggressions/bullying in the workplace

One of the major findings in this survey was the fact that 41% of participants indicated they have experienced microaggressions/bullying in their workplace. Race/ethnicity/religion were the main reasons: this is similarly reflected in the following two studies. In 2021, Alsharif et al.^9^ reported an exploratory study to assess the experience of pharmacy educators of Arabic heritage. As members of the Arab American Pharmacy Educators (AAPE) with EDI in the workplace, the respondents (N = 31) reported experiencing microaggressions and discrimination at work, which is their highest rating on the Likert scale 1 to 5. Some respondents ranked third as perceived racism affecting their performance at work. These results are very similar to the findings of this study, where racism and ethnicity were the two major reasons respondents identified as causes for microaggression. Alsharif et al. stated that being of Muslim religion (pushed to the forefront with 9/11) is impeding professional progression and a perceived source of racism.^9^ The other study investigated the experience of pharmacy students. Avant et al.^10^ explored pharmacy students’ microaggressions and their lived experiences using one-to-one interviews (N = 13). Half were white, with three Black, two Asian and one multiracial student. The dominant theme was the ‘othering feeling’; this aspect was reported by the respondents of this research as well. The students also reported the pervasiveness of microaggressions, often disguised as jokes. Similarly, our study highlighted the same behaviours reported by the respondents. Avant et al.’s^10^ findings highlighted that students in pharmacy, similarly to practising pharmacists, are unsure of how to address and mitigate microaggressions. It is fair to recognise that all healthcare disciplines hold some form of implicit bias, with many of them unaware of its impact on others.

Another prominent feature was being a parent /marital status. These groups of individuals felt like being passed over and not considered for all available opportunities. Looking at the survey respondent's demographic details, we can see that females formed the majority (70%) of participants, which is not surprising as pharmacy workforce globally is dominated by women.^11^ Our results showed that being female, married, having a child/children and of a different ethnical background hugely increases the chances of being discriminated in the workplace. Even though these are the exact protected characteristics defined by the Equality Act 2010 and it is illegal to be discriminated against them by law.^12^

### Unsupportive workplaces

It was interesting to note that a significant proportion of the participants did not perceive their organisation as supportive when they experience microaggressions and bullying. Therefore, it is recommended that organisations invest time and resources to understand the experience of their employees and to implement interventions where needed such as educations and training. Diversity training has been proven successful when utilising personal awareness, intergroup dialogue, and perceptive-taking.^10^ However, some argue that that is not enough and that training in cultural competence and humility is needed as these will allow trainees the reflexivity needed to change behaviour.^13^

Experiential data from frontline healthcare practitioners is imperative in understanding the context of the many facets EDI can take in complex institutions and highly regulated work environments, such as delivering high-quality oncology services. ‘Health care is built on science, but its execution is through, among and between human beings with needs and preferences, fears and insecurities, purpose and aspirations and a wish to something greater’.^14^ The literature review using the terms ‘pharmacy’, ‘pharmacy practice’, or ‘ pharmacist’ and ‘diversity’, ‘equity’, and ‘inclusion’ resulted in articles addressing EDI mostly in pharmacy education (students and academic staff) and are situated in the United States, highlighting the lack of research in this field and need to understand pharmacy professionals’ experiences of EDI, especially in the United Kingdom.

### Patient care

The impact of discrimination and bullying/microaggressions extended to patients who were both affected by it and were the perpetrators. In 2010, The American Cancer Society published a new strategy, demonstrating how knowledge of culture can be used at each stage of cancer care to address patient diversity and ensure health equity. This new strategy requires culturally based education, training, and restructuring of the healthcare system.^15^ The World Health Organisation (WHO) has also made recommendations on what is required to close the inequity gap in cancer care through action on social determinants of health. Seminal works have been published since 2003 by Wilkinson and Marmot on the importance of addressing those factors if any change was to be expected.^16^ There is a need for more exploratory research on the impact of EDI and how care is delivered to patients and how patients, in turn, perceive their care as this relates to their perceived wellbeing and sense of responsibility to social justice. Social justice within the context of pharmacy could be described as an essential moral requisite, encompassing not only fair and equal access to treatments for all patients, but also cultural competence of pharmacy workforce. Social justice in healthcare is currently undergoing a major transformation, as our society recognises the need for dismantling inequity and developing safe, supportive, and inclusive working environments.^17^ Cultural competency is the ability to collaborate effectively with individuals from different cultures, and such competence improves healthcare experiences and outcomes for a diverse patient population. Cultural humility is another concept used in healthcare that underpins respect and understanding of different cultures while recognising our own cultural biases. As ‘healthcare providers, we cannot be competent in another individual/patient's culture, rather, we should strive for humility’.

Despite strategic directions encompassing this aspect, this research addresses the need for more evidence on the lack of EDI in healthcare institutions. Our findings in the pharmacy oncology specialty have highlighted that more needs to be done in training and professional development to address unconscious bias and change behaviours. Experts should lead training provisions with a demonstrated track record of producing transformative outcomes or those who have examined their own ideas and feelings regarding racism and its intersections with other marginalised identity domains.

### Limitations of the study

The reasons for low response rate could only be speculated. Historically, BOPA research subcommittee has indicated that survey response rates fluctuate between 10 and 20% and the topic of the survey very often influences the response rate. Our survey's response rate was 13%, which was within the average response rates. There could be a myriad of reasons behind such a low response rate, such as staff being exhausted from working within already financially and morally stretched NHS, lack of time, having other priorities to attend to and simply becoming disengaged from any additional tasks. Another possible reason for low response rates could be how surveys are being promoted, which is usually via social media and emails, so if the BOPA member has opted out to receive emails and is not particularly active on social media-this could potentially reduce the response rate dramatically, as essentially only those members who have opted in to receive emails from BOPA would be a true representative of a survey sample.

The low response rate also shows an urgent need to educate employees on EDI and raise awareness. Some NHS Trusts are already starting to include EDI training as part of their mandatory training; however, there is a risk that this training will become just another ‘tick box’ exercise, being completed without much thought or effort and often using online delivery. Any cultural change challenges our attitudes and requires mentality shifting and organisations’ understanding that one size does not fit all. Collaborative efforts are required from everyone involved in delivering healthcare services to make sustainable changes in how we provide our services and communicate with patients.

The knowledge gained in the context of pharmacists practising in oncology in the United Kingdom is not intended to create generalisable knowledge as it is discipline-specific. However, health care is provided by humans to other humans with larger systems and structural discrimination through institutional practices and policies that explicitly or implicitly differentially impact, or harm, non-dominant groups.
